# Metabolic Control of Epilepsy: A Promising Therapeutic Target for Epilepsy

**DOI:** 10.3389/fneur.2020.592514

**Published:** 2020-12-08

**Authors:** Yanqing Fei, Ruting Shi, Zhi Song, Jinze Wu

**Affiliations:** ^1^Department of Neurology, The Third Xiangya Hospital, Central South University, Changsha, China; ^2^Department of Rehabilitation, The Third Xiangya Hospital, Central South University, Changsha, China

**Keywords:** epilepsy, energy metabolism, brain, ATP-sensitive potassium channel, anticonvulsants

## Abstract

Epilepsy is a common neurological disease that is not always controlled, and the ketogenic diet shows good antiepileptic effects drug-resistant epilepsy or seizures caused by specific metabolic defects via regulating the metabolism. The brain is a vital organ with high metabolic demands, and epileptic foci tend to exhibit high metabolic characteristics. Accordingly, there has been growing interest in the relationship between brain metabolism and epilepsy in recent years. To date, several new antiepileptic therapies targeting metabolic pathways have been proposed (i.e., inhibiting glycolysis, targeting lactate dehydrogenase, and dietary therapy). Promising strategies to treat epilepsy via modulating the brain's metabolism could be expected, while a lack of thorough understanding of the role of brain metabolism in the control of epilepsy remains. Herein, this review aims to provide insight into the state of the art concerning the brain's metabolic patterns and their association with epilepsy. Regulation of neuronal excitation via metabolic pathways and antiepileptic therapies targeting metabolic pathways are emphasized, which could provide a better understanding of the role of metabolism in epilepsy and could reveal potential therapeutic targets.

## Introduction

Epilepsy is a brain disease with neurobiological, cognitive, psychological, and social consequences, characterized by an enduring pre-disposition to generate epileptic seizures (1). Regardless of its etiology, epilepsy is widely regarded as a disease of neuronal network excitability unbalance from altered ionic or synaptic transmission (2). Excessive synchronized discharge of neuronal networks causes epileptic seizures and is a specific manifestation of network excitability changes, such explosive electrical activity, that must be supported by enhancing metabolism (3, 4).

Most activities involve and are influenced by metabolism. Numerous individuals have various diseases caused by inborn or acquired metabolic dysfunctions. Over 500 inborn errors of metabolism were confirmed and affected ~1 in 2000 live births (5). Except for some typical metabolic endocrine diseases, many acquired diseases could involve systemic or focal metabolic changes that could be helpful in diagnosis or prognosis and as therapeutic targets (6–8). It is well-recognized that human health is strongly related to metabolism, but this has only started to attract sufficient attention in the last decade. Scientific developments have led to an in-depth understanding of metabolism and its role in pathophysiological processes. Metabolic changes both accompany diseases and can comprise therapeutic targets. Hippocrates first documented calorie restriction therapeutics to treat epilepsy (9). Based on this, the ketogenic diet emerged (10) and has played an important role in antiepileptic therapy (see section Dietary Therapy in Epilepsy).

Recently, neuroscientists have proposed several antiepileptic treatment methods that involve metabolic regulation (11–13). Technological developments have facilitated the detection of metabolic changes; then, epilepsy foci can be located (14). Today, researchers have acquired a larger understanding of the brain's metabolism and its roles in epilepsy, and recent findings have shown that there is a significant association between epilepsy and metabolism, with some researchers defining epilepsy as a *metabolic disease* (2, 15, 16). However, the relationship between metabolism and epilepsy has rarely been examined in detail. This review focused on the relationship between metabolism and epilepsy aiming to provide a better understanding of the role of metabolism in epilepsy and to reveal potential therapeutic targets.

## Metabolic Features in the Brain

It is an established fact that the brain is the most developed part of the nervous system, controlling nearly all activity and adjusting the body to the external environment. Although the adult human brain accounts for only 2% of body weight, it consumes 20% percent of the body's oxygen. Interestingly, the mass of a child's brain (5-year-old) accounts for 6% of body weight and consumes 50% of the body's oxygen (17). Additionally, 12% of the cardiac output will flow to the brain (18). It was estimated that adenosine triphosphate (ATP) consumption in the gray matter of the brain is 30 mmol ATP/Kg tissue/min, which is close to the use of muscles in the human leg during a marathon (19). These data suggest that the brain has a stunning energy metabolism. While the metabolic properties of the brain are not limited to these, some unique metabolic characteristics are closely relevant to epilepsy treatment.

### Glycolysis Is Essential for Brain/Neuronal Function

Glucose is the most important energy source in the brain, with some additional energy substrates having been reported, e.g., ketones and lactate (20, 21). These alternative energy substrates substitute glucose when glucose deficiency occurs, but they can only partially compensate for glucose. Evoked population spikes were attenuated by decreasing the glucose levels in a culture medium even without altering the intracellular concentrations of ATP or phosphocreatine (PCr); abnormal synaptic function could not fully recover by replacing glucose with pyruvate, lactate, or other energy substrates (22). This evidence showed that decreased glycolysis due to insufficient glucose concentration impairs neuronal function, and glycolysis is crucial for sustaining synaptic function. Conversely, to support increased synaptic vesicle circulation, the glycolysis levels in activated neurons were significantly increased (2-fold change from baseline), especially in the pre-synaptic terminals (23). These results may have been derived from brain slices but are suitable for the entire brain. Glucose does not consume oxygen in anaerobic glycolysis, while 1 mol of glucose consumes 6 mol of oxygen gas (*O*_2_) in aerobic oxidation: 1 *glucose* + 6*O*_2_ = 6*CO*_2_ + 6*H*_2_*O*. The ratio of *O*_2_/glucose consumed by the brain is called the oxygen–glucose index (OGI) and is maintained at a value close to six during rest, until stimulation leads to metabolic changes. Even if sufficient oxygen is delivered to the brain, the OGI decreases during brain activation by several types of stimulation, such as vigorous motor and complex cognitive tasks or by pathological conditions (seizures and depression). This marks a preferential increase in glycolysis in the activated brain, even if oxygen availability is sufficient (17). Furthermore, this may account for the antiepileptic effect of glycolysis inhibition (see section Inhibiting Glycolysis to Reduce Seizures).

### Astrocyte-Neuron Lactate Shuttle (ANLS)

The cerebrum is composed of ~100 billion neurons and one trillion neuroglia cells. Astrocytic endfeet are a fundamental and important component of the blood–brain barrier (BBB); almost 99% of the surface of blood capillaries are enwrapped by astrocytes end feet (24), signifying that most of the neurons in the brain do not directly contact the capillaries. Then, how could neuronal energy uptake substrates and other nutritional materials sustain a high-intensity metabolism become a question. Although the classical theory holds that glucose is the main energy source for neurons, new viewpoints are emerging. In 1994, Pellerin and Magistretti first proposed the mechanism of ANLS (25) that has been frequently discussed in recent years and may thoroughly explain the neuroenergetics at the cellular and molecular levels.

**Figure 1:**
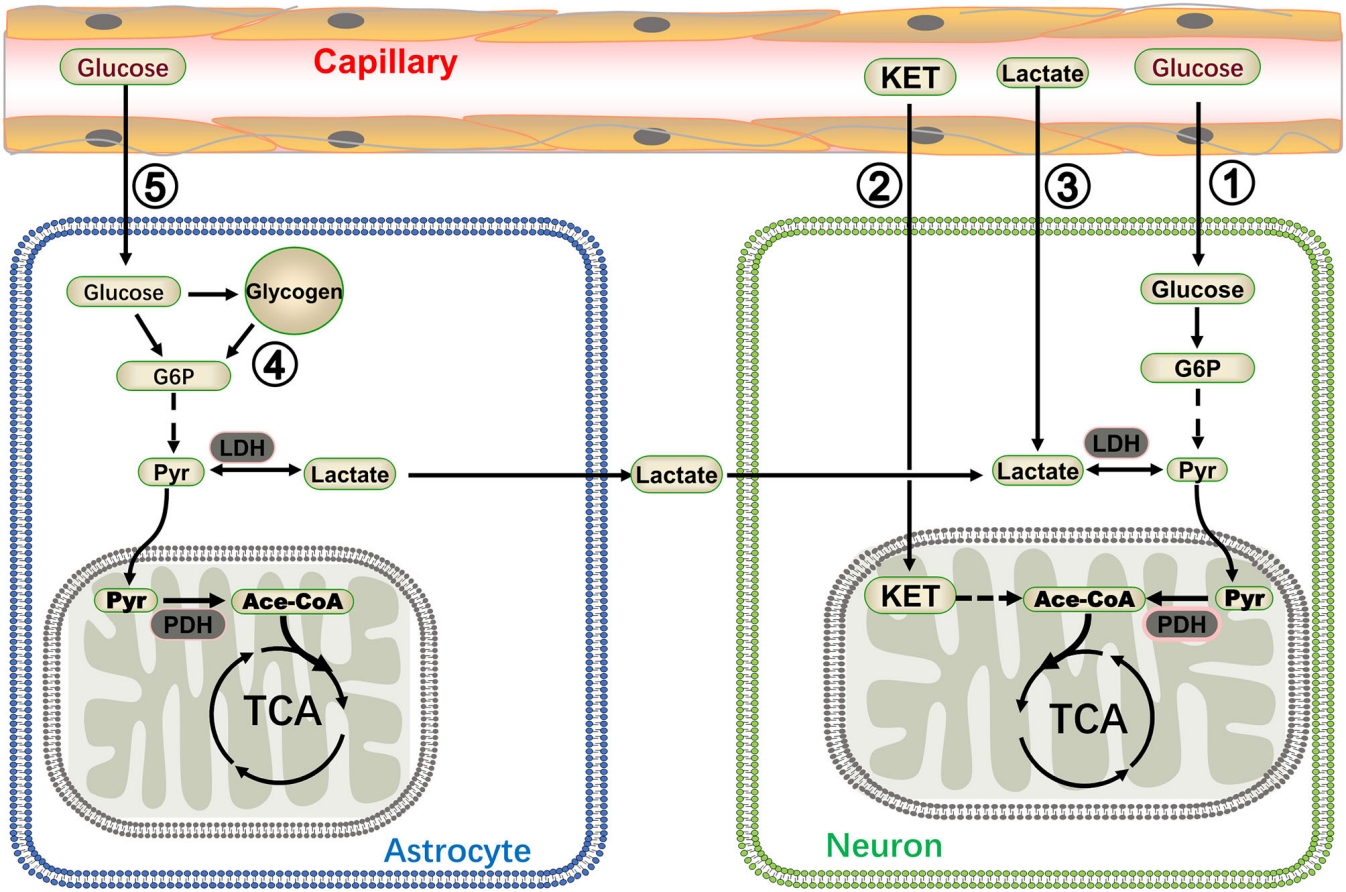
A summary of energy metabolism patterns in neurons. Glucose and ketone (KET) are recognized as energy substrates, which are labeled as ① and ② respectively. Lactate in the blood can also be utilized as an energy source for the brain during moderate or intense exercise (marked as ③) (20, 29). Glycogen stored in astrocytes generated lactate via glycogenolysis and lactate was then shuttled to neurons (marked as ④) (30). The astrocyte-neuron lactate shuttle is very important in sustaining the energy metabolisms of neurons during high synaptic activity (marked as ⑤) (13). G6P: glucose 6 phosphate, Pyr, pyruvate; PDH, pyruvate dehydrogenase; Ace-CoA, acetyl-coenzyme A.

Most neurons in the brain do not directly contact the capillaries; astrocytes are employed as a bridge to connect the capillaries and neurons and transport energy substrates. Glucose is absorbed by astrocytes through glucose transporters from the capillaries or extracellular fluid, and then translated to lactate via glycolysis. Lactate was traditionally regarded as a waste product of glycolysis. However, new studies have indicated that lactate is an important energy substrate for normal tissues and/or tumors (26, 27); in the brain, lactate produced in astrocytes fuels the mitochondrial tricarboxylic acid (TCA) cycle of neurons in a proprietary way, i.e., the ANLS (28). According to the ANLS, astrocytes absorb glucose and convert it to lactate. Then, lactate is transferred out of the astrocytes through the type 1 and 4 of monocarboxylate transporter (MCT1, 4) and carried into the neighboring neurons through MCT2. Lactate originating from the astrocytes is again catalyzed to pyruvate by lactate dehydrogenase, and the pyruvate could be carried into the mitochondria and be utilized as an energy metabolite in the Krebs cycle (Figure 1). Although this theory remains controversial, the concentration of lactate in astrocytes was significantly higher than that in neurons, and this lactate gradient provided a pre-condition for the flow of lactate from astrocytes to neurons mediated by carriers (31). Furthermore, the ANLS theory makes perfect use of the astrocytes' support function; it reduces neuronal dependence on glycolysis, i.e., a multifarious biochemical reaction process that produces a very small amount of ATP. Hence, neurons can derive more energy with as few biochemical steps as possible (17). This pattern of energetics mainly exists in activated excitatory neurons and is very important in sustaining the energy metabolism of neurons during high synaptic activity; therefore, it may be involved in certain diseases, such as epilepsy (32). Targeting the LDH-a key enzyme in the ANLS exhibited an antiepileptic effect (13) (see section Targeting Lactate Dehydrogenase to Treat Epilepsy).

### Various Energetics in the Brain

Organisms can survive in various complex environments because organs can change their metabolism for adaptation purposes. Glucose in plasma or extracellular fluid remains the most important energy substrate for the brain, with numerous other substrates fueling the brain via various pathways. Glycogen stored in the liver or muscles is an important alternative energy source for the human body. It is also present in human brain tissue (33) and plays a crucial role in memory formation, learning capacity, and regulation of the sleep-wake cycle (34–36). As a matter of fact, glycogen is an energy reserve selectively localized in astrocytes (37). It is lactate that fuels the neurons through the ANLS and plays important roles in the brain. In some special circumstances, such as high-intense memory tasks, sleep deprivation, and hypoglycemia, lactate derived from astrocytic glycogen fuels the brain during exhaustive conditions (30). In general, blood glucose could contribute to brain energetics, and lactate in the blood can also be utilized as an energy source for the brain during moderate or intense exercise (20, 29). In addition, ketone bodies comprise an important alternative energy substrate for the brain and could be directly utilized to maintain the energy metabolism in a starvation condition or when following a ketogenic diet (21). Various energy supply modes enable the nervous system to adapt to various states and offer the potential to better understand and treat diseases (the energy metabolism in the brain is illustrated in Figure 1).

## Changes in Metabolic Characteristics in Epilepsy

There are often metabolic changes in a diseased organ or tissue. Sometimes, these metabolic changes contribute to diagnosis or treatment, which could be applicable to epilepsy. Similar to the energy changes associated with earthquakes, epileptic foci are often accompanied by significant energy metabolism changes during seizures (4, 38). With the development of detection methods and technological progress, evidence supporting this point is accumulating.

### Neuroimaging

The lesions caused by general diseases can be found by CT or MRI, while the location of epilepsy lesions is more complicated. Sometimes, epileptic lesions do not show structural abnormalities, but can be detected through differences in metabolic levels or combined with electroencephalography (EEG) to locate the epileptic foci. ^18^F-FDG positron-emission tomography (PET) makes it possible to intuitively observe the metabolic differences in brain regions and locate the epileptogenic foci. PET is usually used as interictal investigation, and epileptic foci show characteristic hypometabolism in the inter-ictal phase (39). PET scanning is almost impossible to perform in epileptic patients with motor symptoms during the seizure period, but it can be used for absence epilepsy. During the ictal phase of absence epilepsy, the metabolism of the thalamus is obviously enhanced, and cerebral blood flow is increased in the whole brain (40–42). Animal experiments have supported that there is enhancement of brain metabolism during epileptic seizures. In a rat seizure model induced by pilocarpine, FDG-PET imaging showed significant hypermetabolism in the area of the hippocampus and entorhinal cortex during status epilepticus (43). Single-photon emission computed tomography (SPECT) also constitutes a technique to evaluate brain metabolism and to locate epileptic foci. SPECT imaging can monitor dynamic changes in cerebral perfusion, thereby reflecting the metabolic changes before, during, and after seizures. True ictal SPECT imaging has shown that hyperperfusion emerges in the epileptogenic region, and indirectly reflects increased brain metabolism through changes in cerebral perfusion (44).

### Biochemical Changes in Epilepsy

Epilepsy manifests as a change in consciousness and/or behavior, with abnormal EEG activities being at the root of seizure causality. The electrical activity must be accompanied by changes in energy; in fact, the conversion between substances will produce energy changes. Therefore, epilepsy must be accompanied by changes in energy-related substances; some abnormal neurochemical changes occur in the epileptic brain. However, elevated peripheral blood lactate levels are considered to be correlated with the extent of disease or injury (45). Comparing with the non-epileptogenic hippocampus, the concentration of lactate in the epileptogenic hippocampus increased from 4.6 ± 0.4 to 6.8 ± 0.7 mM (*P* < 0. 001) (46–48). An increase in anaerobic glycolysis was also shown in the epileptogenic brain. In addition, status epilepticus causes a significant increase in cerebrospinal fluid (CSF) lactate, and the magnitude of lactate elevation could play an important role in predicting the morbidity and mortality of status epilepticus (49).

## Metabolism Dysfunction Leads to Epilepsy

We so far discussed the metabolic characteristics of the brain and energy metabolism changes in epilepsy. Additionally, metabolic disorders can lead to epilepsy. Next, we introduce several types of common epileptic diseases, including the glucose transporter (GLUT1) deficiency syndrome, hypoglycemia, creatine deficits, and mitochondrial encephalomyopathies.

### GLUT1 Deficiency Syndrome

As discussed above, the brain is an important organ with high energy demand and glucose uptake via GLUT1 on the endothelial cells of the BBB. Although there are several subtypes of GLUTs in the brain, mainly including GLUT1 on the endothelial cells of the BBB, GLUT2 on astrocytes, and GLUT3 on neurons (50, 51), GLUT1 is the one most importantly associated with epilepsy (52). Defects of GLUT1 will impair glucose transport into the brain and result in Glut-1 deficiency syndrome (53). Glut1 deficiency syndrome is an autosomal dominant hereditary neurologic disorder characterized by low glucose (<40 mg/dL) and low lactate levels in the CSF. Seizures, which often initiate in the first 4 months of life, are the most common presenting symptom in this disorder. Patients also often have stunting, acquired microcephaly, ataxia, and muscle spasms. Sudden onset of confusion, lethargy, sleep disturbances, and headaches may also occur. The extent of cognitive impairment could vary with the condition. Epilepsy caused by Glut1 deficiency syndrome shows different types: complex-partial seizures (53), absence epilepsy (54), generalized tonic-clonic seizures (55), and others. In addition, mutations in GLUT1 (also known as SLC2A1) can also cause a syndrome called focal epilepsy (FE) and paroxysmal exercise-induced dyskinesia (PED) (56), whose attacks may be associated with increased glucose consumption caused by exercise. Ketogenic diet therapy is the gold standard treatment for patients with GLUT1 deficiency syndrome (57). The age at the correct diagnosis is the most important factor determining prognosis, and early diagnosis is very important as well as initiating the ketogenic diet as soon as possible (58).

### Hypoglycemia

Hypoglycemia is a common, acute life-threatening illness in diabetic patients that may cause seizures. Not all patients with hypoglycemia have epileptic seizures; neonates and children appear to be more susceptible to epileptic seizures induced by hypoglycemia, which may be associated with brain immaturity (59). Furthermore, hypoglycemia-induced seizures may be linked to genetic susceptibility. In the background of DBA mice prone to epileptic seizures, insulin injection that reduced blood sugar levels to 60%, increased spike-and-wave discharge (SWD) by >300%, whereas the same dose of insulin could not induce SWD events in C57Bl6 mice with epileptic seizure resistance (60). This difference decided by genetic susceptibility has not been confirmed in the population, but patients with systemic epilepsy are more sensitive to hypoglycemia; Increased cortical excitation was much more obvious in epileptic patients than in healthy individuals under starvation conditions, which indicated that the neuronal networks in the epileptic brain were more susceptible to hypoglycemia (61). The mechanism of this sensitivity difference is not clear at present. In general, most epileptic seizures caused by hypoglycemia can be quickly relieved after blood glucose level recovery. However, attention is required in that children with diabetes with hypoglycemic convulsions could eventually develop perpetual abnormal EEG. Good control of blood glucose did not improve the abnormal EEG caused by hypoglycemia. Severe hypoglycemia in infantile or early-onset diabetes mellitus appears to be an important risk factor for persistent EEG abnormalities (59). Hence, the prevention of hypoglycemia is very important, especially in infants.

### Creatine Deficits

Creatine deficiency syndrome comprises a group of disorders caused by creatine (Cr) synthesis or transport defects. The main symptoms of creatine deficiency syndrome include intellectual impairment, severe language delay, behavioral abnormalities, and seizures (62). Creatine is the most important material for the synthesis of the high energy compound, creatine phosphate. L-arginine glycine amidine transferase (AGAT) and guanidine acetate methyltransferase (GAMT) are key enzymes in creatine synthesis. Creatine synthesized in the liver is transported by creatine transporter 1 (CT1) to muscle and brain tissues with high energy metabolism (63). Hence, deficits in the synthesis or transport of creatine can lead to creatine deficiency. The biosynthesis dysfunction of creatine comprises two autosomal recessive disorders: AGAT deficiency and GAMT deficiency.

AGAT deficiency is a rare disease with only individual cases having been reported and involves non-specific symptoms including intellectual disability and epilepsy (63). Various GAMT mutations have been reported (64, 65). GAMT deficiency can lead to severe early epileptic encephalopathy and a range of developmental, behavioral, and motor disorders. Approximately 50% of patients develop seizures, which are the second most common symptom in GAMT deficiency. The most common types of epilepsy reported include febrile convulsions, generalized tonic-clonic seizures, and myoclonic seizures (63). CT1 defect is a relatively common X-linked disease due to SLC6A8 mutation. Intellectual disability and epilepsy are common in CT1 defect, and the types of seizures include febrile, myoclonic, generalized tonic-clonic seizures; convulsive status epilepticus, and partial seizures with secondary generalization (63, 65). Oral creatine supplementation was shown to effectively improve the clinical symptoms of disorders of creatine biosynthesis (66). Treatment for GAMT deficiency that corrects creatine depletion and reduces the accumulation of the enzyme product guanyl acetic acid (GAA) can be successful (64, 67). Most evidence suggests that creatine supplementation is not effective in patients with CT1 defect, and conventional antiepileptic drugs could control well epilepsy in CT1 defect (68).

### Mitochondrial Encephalomyopathies

Traditionally, mitochondrial diseases refer to a group of hereditary disorders in which mitochondrial DNA (mtDNA) or nuclear DNA (nDNA) deficiencies cause oxidative phosphorylation dysfunction of the mitochondrial respiratory chain (69). Mitochondrial encephalomyopathies are the most serious mitochondrial disease involving the brain and muscles. Myopathy, encephalopathy, lactate acidosis, and stroke-like episodes (MELAS), myoclonus epilepsy and ragged red fibers (MERRF), and polymerase gamma (POLG)-related disease are the three most common mitochondrial encephalomyopathies and are closely associated with epilepsy (70). In MELAS, epilepsy is one of the most frequent and early onset symptoms that mainly occurs in the group of patients with stroke-like lesions, and seizures are often accompanied by migraine-like headaches. Patients with MELAS are prone to status epilepticus, which may be the first symptom (70). Just as its name implies, patients with MERRF are usually characterized by progressive myoclonic seizures, and epilepsy in most patients tends to develop to generalized tonic-clonic seizures (71). As for POLG-related disease, it is caused by POLG defect and related to Alpers-Hüttenlocher syndrome or to adult-onset encephalopathy, spinocerebellar ataxia, and epilepsy (72). Myoclonic seizures are a common and obligatory feature in both multisystemic disorders, and epilepsy in POLG-related disease is often resistant to drug therapy (73).

### Possible Mechanism of Seizures Caused by Metabolism Dysfunction

A common characteristic of these relatively widespread metabolic disorders leading to epileptic seizures is a decrease in energy substances, with the ultimate result being a decrease in ATP levels. Continued severe energy crisis could lead to epileptic seizures. The possible mechanisms are as follows. First, the resting potential of neurons determines neuronal excitation. The sodium-potassium pump (Na^+^-K^+^ ATPase) plays an important role in retaining the resting potential through ATP consumption. The function of the sodium-potassium pump will become impaired when ATP produced in neurons decreases, which will lead to a decrease in the absolute value of the resting potential. The relative depolarization of neurons increases excitability and leads to epileptic discharges (74). Second, neural networks in the hippocampus can be inhibited by the activation of intermediate neurons by purine receptors. ATP reduction may reduce the inhibition effect, facilitating the spreading of excitation in the neural network (75, 76). Furthermore, Na^+^-K^+^ ATPase dysfunction reduces the GABAergic potentials, thereby enhancing the excitatory post-synaptic potentials and spike firing leading to reduced inhibition and increased excitation; this is also an important mechanism underlying hyperexcitability and is associated with increased epilepsy sensitivity (77). Therefore, energy metabolism dysfunction will lead to epileptic seizures, especially for energy deficiency in normal cells.

## Metabolites Affect Neuronal Excitability

Epilepsy can cause systemic or focal metabolic changes, and some metabolic disorders can lead to seizures. It is the metabolites that affect the excitability of neurons. Next, we will introduce the effects of two important metabolites on neuronal excitation.

### Lactate

**Figure 2:**
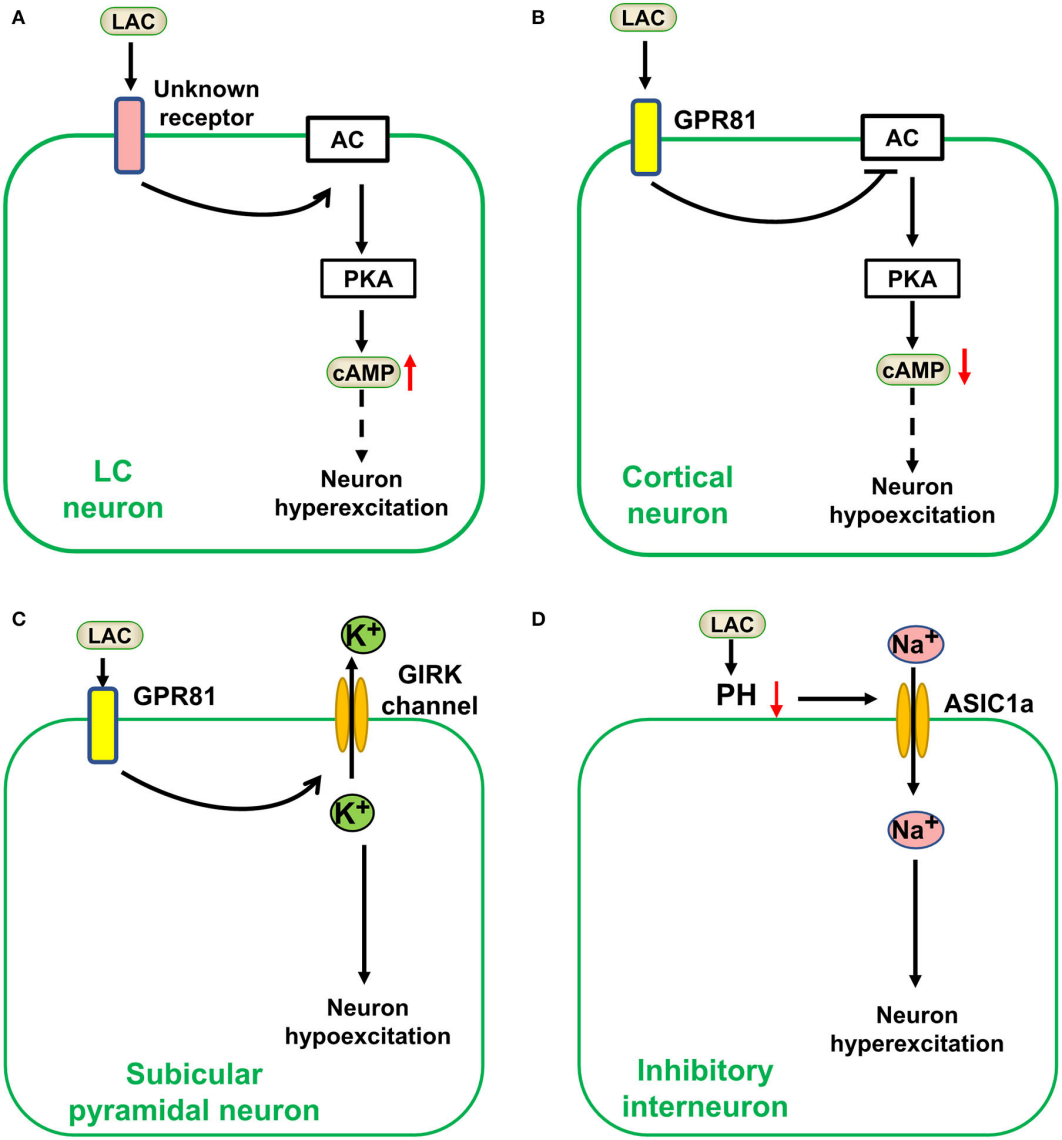
Lactate affects neuronal excitation as a signal molecule in the brain. Lactate (LAC) affects neuronal excitation as a signal molecule in the brain. **(A)** Exogenous lactate was excitatory to locus coeruleus (LC) neurons via an unknown receptor-mediated pathway (80). **(B)** Lactate over physiological levels acted as an inhibitor for cortical neurons a GPR81-mediated pathway (81–83). **(C)** Exogenous lactate (over physiological levels) induced an outward current mediated by G protein–gated inwardly rectifying potassium (GIRK) through activating GPR81, resulting in hyperpolarization and epileptiform firing decrease in the subicular neurons (84). **(D)** Lactate could regulate acid-sensing ion channel−1a (ASIC1a) by decreasing pH to Stimulate inhibitory neuronal excitation (85). AC, adenylate cyclase; PKA, protein kinase A; cAMP, cyclic adenosine monophosphate.

Traditionally, lactate was considered a metabolic waste produced by anaerobic glycolysis and a sign of the severity of some diseases (49). However, a recent study suggested that lactate is the energy substrate of some cancer or normal tissue cells, and its priority may be higher than glucose (26). In the past two decades, lactate was in the spotlight, as researchers focused on its metabolic role in the brain. Besides this still debated metabolic role, lactate could act as a signal molecule in brain cells (78). In 2008, lactate was found to be a natural ligand for the hydrocarboxylic acid-1 receptor [HCAR1, also named G protein-coupled receptor (GPR) 81 receptor], a GPR primarily regulating the cAMP signaling pathway (79). Thus, the possibility that lactate plays a physiological and pathological role through G protein-coupled receptors was greatly expanded. Moreover, increasing numbers of studies have shown that lactate is a by-product of metabolism and an important signal molecule in regulating neuronal excitation. However, its effects on neuronal excitation in different brain regions differ, even being completely opposite. Although exogenous lactate was excitatory to locus coeruleus neurons, further research showed it may act through an unknown receptor or pathway; however, it is highly unlikely that GPR81, which was described in the adipose tissue previously, could be responsible for the effects on LC neurons induced by lactate (Figure 2A) (80). Lactate over physiological levels acted as an inhibitor for cortical neurons as it inhibited the excitability of cortical neurons through a metabolic or a GPR81-mediated pathway (Figure 2B) (81–83). Exogenous lactate may also act as an inhibitor in hippocampal neurons. According to a recent research, exogenous lactate induced outward currents mediated by G protein–gated inwardly rectifying potassium(GIRK) through activating GPR81, resulting in hyperpolarization and epileptiform firing decreasing in the subicular neurons of hippocampal slices (Figure 2C) (84). Furthermore, lactate could regulate some ion channels by decreasing pH. Lactate increases TREK1 channel activity by reducing intracellular pH (86), and reduced the damage to neurons caused by hyperexcitation. However, acid-sensing ion channel-1a (ASIC1a) is sensitive to extracellular pH and regulates neuronal excitation (Figure 2D). It was revealed that ASIC1a on inhibitory interneurons is activated by reducing extracellular pH and could terminate seizures (85). Lactate as a substrate for energy metabolism affects neuronal excitation. The latter will be explained in section Control of Epilepsy by Regulating Metabolic Pathways.

In sum, lactate can affect neuronal excitation as a signal molecule. However, its physiological concentration is not sufficient to activate the known GPR81 receptor (78) and its direct effect on epilepsy as a signal molecule is uncertain.

### ATP

ATP is the universal energy currency of life. However, it is an important signaling molecule involved in apoptosis (87) and autophagy (88). ATP in the nervous system is involved in ischemia, epilepsy, Parkinson's disease, infection, amyotrophic lateral sclerosis, Alzheimer's disease, etc (89). As a signal molecule, ATP plays an important role in affecting neuronal excitation through intracellular or extracellular pathways. The intracellular pathway regulates the excitability of neurons mainly by regulating the opening of ATP-sensitive potassium (K_ATP_) channels. A K_ATP_ channel opens when ATP levels are decreased, potassium ion outflows, and the cell membrane becomes hyperpolarized, leading to neuronal excitation decrease (90). Studies have shown that epilepsy treatment by modulating metabolism mainly acted through this intracellular ATP/K_ATP_ signaling pathway (13, 91). Regarding the extracellular pathway, ATP derived exogenously or from glia is a widely distributed cell-to-cell signaling molecule in the brain. It is the endogenous ligand of purine receptors (P2Xs, P2Ys). The P2XRs receptors may mediate chronic neuromodulation of the entire nervous system by acting on glia, especially on astrocytes (92). Thus, the scope of its impact is larger, and its specific role is unclear. While the role of ATP in activating P2Ys receptors is clear, extracellular ATP could activate the intermediate neurons mediated by P2Y1 receptors in the hippocampus (76). Therefore, extracellular ATP acts as an inhibitor in the hippocampal neuronal network (75). Theoretically speaking, activation of interneurons contributes to inhibition of the excitability of the neural network and, thus, reduces seizures, but the anti-epileptic effect of targeting purine receptors is uncertain. Simultaneously, extracellular ATP is unstable and easy to be decomposed to adenosine (93). In terms of extracellular pathways, ATPergic signaling is complex and its role in epilepsy remains unclear.

## Control of Epilepsy by Regulating Metabolic Pathways

Epilepsy treatment by inhibiting metabolism may originate from the antiepileptic effect of the ketogenic diet, which mimics a state of starvation or calorie restriction and regulates neuronal excitation via energy metabolism modulation (94, 95). Therefore, avenues of treating epilepsy by targeting metabolism are opened.

### Inhibiting Glycolysis to Reduce Seizures

**Figure 3:**
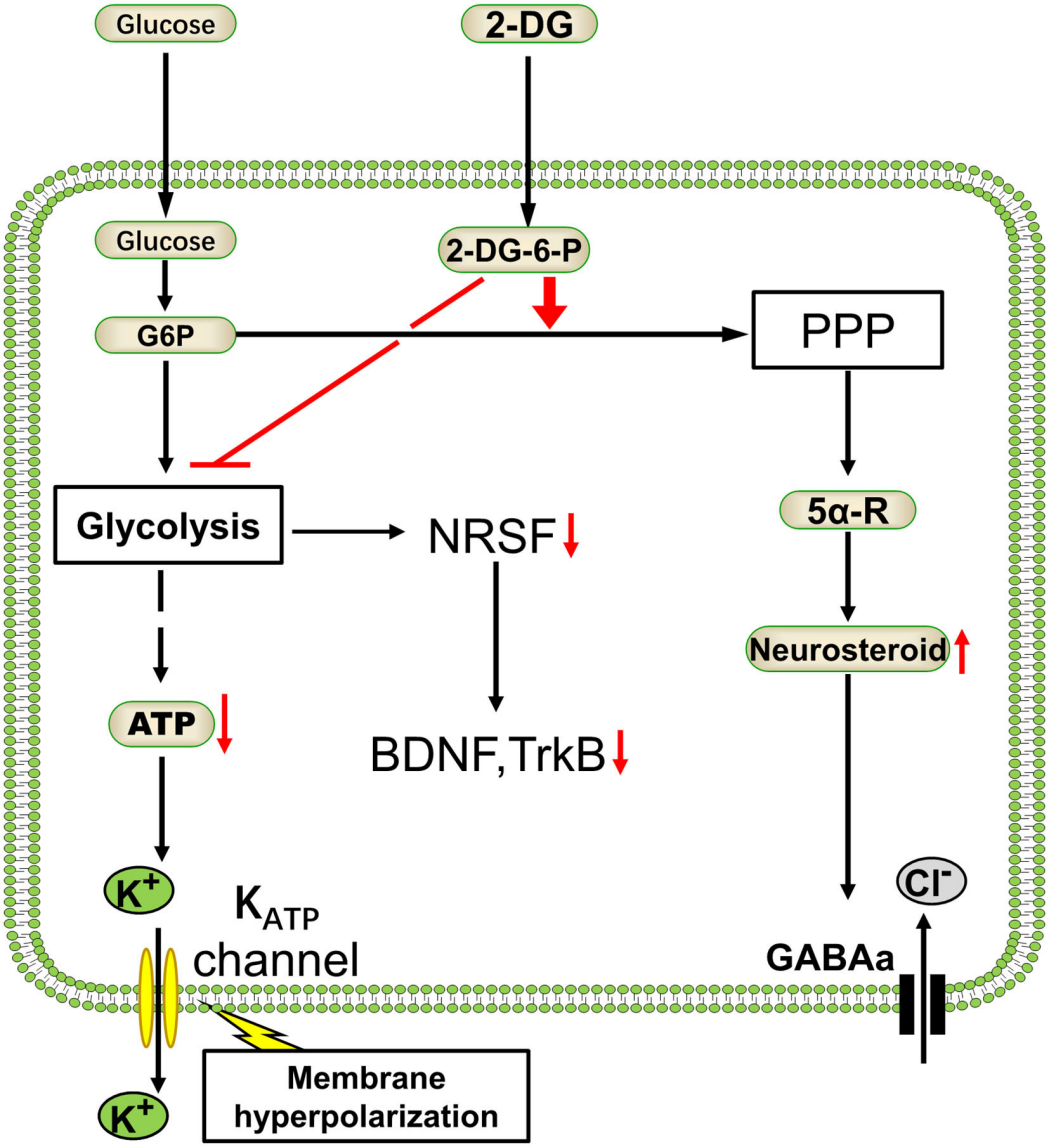
Antiepileptic mechanism of glycolysis inhibitor 2-deoxyglucose. As a classical glycolysis inhibitor, 2-deoxyglucose (2-DG) would inhibit the energetics of glucose and decrease the ATP. Glycolysis reduction will enhance the pentose phosphate pathway (PPP) metabolism and resulting in GABAergic strength (91). On the other hand, 2-DG reduces epilepsy progression by NRSF–dependent metabolic regulation of BDNF and TrkB (11). Glc, glucose; G-6-P, glucose-6-phosphate; 2-DG-6-P, 2-deoxyglucose-6-phosphate; NRSF, neural restrictive silencing factor; BDNF, brain-derived neurotrophic factor; TrkB, tyrosine kinase receptor B; 5α-R, 5α-reductase.

In section Glycolysis Is Essential for Brain/Neuronal Function, we discussed the importance of glycolysis in maintaining neuronal function. Glycolysis may preferentially increase when the brain is in a certain pathological state, such as seizures. Inhibition of glycolysis reduces abnormal neuronal activity. 2-Deoxy-d-glucose (2-DG), a typical glycolysis inhibitor that can significantly reduce glucose uptake and glycolysis (96), has exhibited antiepileptic effects in several animal models (11, 91). The mechanisms of glycolysis inhibitor 2-DG in reducing epileptic seizures vary. First, 2-DG could activate the K_ATP_ channel (91), an inward rectifier potassium channel, which is sensitive to and regulated by intracellular ATP concentration. High and low ATP concentration in the cytoplasm inhibits and opens K_ATP_ channels, respectively, resulting in hyperpolarization of the neuronal cell membrane by potassium ion outflow and decreasing neuronal excitation. Thus, it plays a role in inhibiting epilepsy (91, 97). Second, in a rat kindling model of temporal lobe epilepsy, 2-DG reduced seizure progression by reducing the expression of brain-derived neurotrophic factor (BDNF) and its receptor, TrkB; this reduced expression is mediated by the decreasing of transcription factor NRSF caused by 2-DG administration (11). Previous studies have shown that conditional TrkB knockout (98) or BDNF signaling pathway reduction through transgenic technology (99) could reduce seizures in kindled animal models. These mechanisms can recur neuronal circuits that promote hyperexcitability (100), thus reducing the BDNF pathway, may have antiepileptic effects. In addition, 2-DG promotes the production of nicotinamide adenine dinucleotide phosphate (NADPH) by enhancing pentose phosphate pathway (PPP) metabolism in cells. The higher concentration of NADPH will potentiate the biosynthesis of neurosteroids via enhancing the activity of 5α-reductase (5α-R), a crucial enzyme catalyzing the steroid precursors into neurosteroids (101), resulting in potentiating the GABAergic tonic inhibition (91, 101) (Figure 3).

The antiepileptic effect of 2-DG by inhibiting glycolysis has also been confirmed by other studies. The Bcl-2-associated death promoter (BAD) is a member of the BCL-2 family with dual functions in proapoptosis and glucose metabolism. Gene knockout or alteration of BAD will impair glucose metabolism in brain cells and activate the ATP-sensitive K_ATP_ channels on the neuronal membrane, leading to an induced resistance to seizures (12, 102). A high dose of Fructose-1,6-Bisphosphate (F1, 6BP) was also demonstrated to suppress seizures in animals [(103–106)]. F1, 6BP becomes an inhibitor of PFK1, a rate-limiting enzyme in glycolysis. Therefore, excessive exogenous F1,6BP may play an inhibitory role in neurons (103). In conclusion, glycolysis inhibition, such as by 2-DG, is a promising treatment for epilepsy [(107, 108)].

### Targeting Lactate Dehydrogenase to Treat Epilepsy

**Figure 4:**
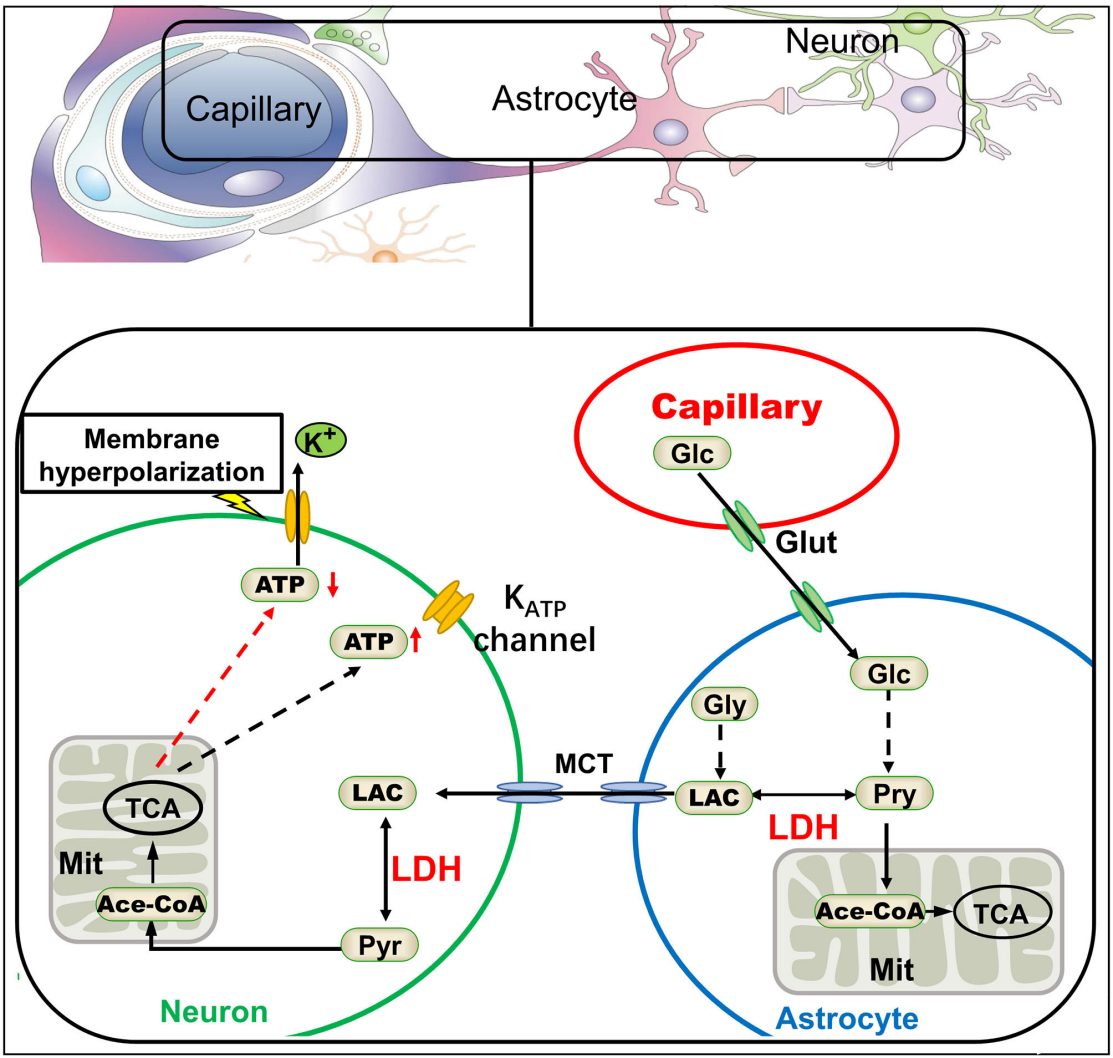
The astrocyte-neuron lactate shuttle and antiepileptic mechanism of LDH inhibition. According to the astrocyte-neuron lactate shuttle, lactate dehydrogenase (LDH) plays a key role in the energy supply of neurons. Inhibition of LDH would lead to the decrease of ATP in neurons, thus activating KATP channels on the neural membrane and potassium efflux, finally making the neural membrane hyperpolarization (13). Glc, glucose; Glut, glucose transporter; Pyr, pyruvate; Gly, glycogen; Lac, lactate; TCA, tricarboxylic acid cycle; MCT, monocarboxylic acid transporter.

Lactate dehydrogenase (LDH), one of the most abundant proteins in the cell cytoplasm, is a type of enzyme widely existing in tissues. LDH is known as a biomarker of disease and tissue damage (109, 110). Increased LDH activity was found in some epileptic kindling models (111). It catalyzes the mutual conversion between lactate and pyruvate. Pyruvate is the final product of aerobic glycolysis and generates ATP in the mitochondria. Presently, lactate is no longer regarded as metabolic waste, as it fuels various tissues even under fully aerobic conditions (26, 27). Lactate in the brain acts as an energy substrate through the ANLS (discussed in section Metabolic Features in the Brain). Glucose derived from glycogenolysis or peripheral circulation is metabolized by glycolysis to produce pyruvate or lactate. Pyruvate, which is the end product of glycolysis, can be utilized by the mitochondria of astrocytes to generate ATP. Conversely, it can be transformed into lactate by LDH. Then, the lactate produced in astrocytes is shuttled from astrocytes to the adjacent neurons by monocarboxylic acid transporters (MCTs). After shuttling into the neurons, lactate is again converted to pyruvate by LDH, and pyruvate could be used by the mitochondria to feed the tricarboxylic acid cycle. ATP is produced through oxidative phosphorylation within the mitochondrial respiratory chain (MRC), and then released into the cytoplasm (45). LDH plays a crucial role in this pathway, and its inhibition will interfere with the ANLS to decrease ATP production in neurons. A high concentration of ATP in the cytoplasm inhibits K_ATP_ channels, while K_ATP_ channels open when intracellular ATP concentration decreases, resulting in hyperpolarization of neuronal-cell membrane by potassium ion outflow and decreasing neuronal excitation. Thus, LDH inhibits neuronal discharges and plays a role in inhibiting epilepsy (97) (Figure 4). Inhibition of LDH by oxamate had been demonstrated in mice. Another study found that stiripentol was also an LDH inhibitor, and LDH inhibition may be one of its important antiepileptic mechanisms (13). Interestingly, the mTOR pathway, i.e., an important signaling pathway involved in epilepsy, was also inhibited by LDH inhibitor oxamate (112, 113). In addition, activation of the mTOR pathway increased the expression of LDH (114, 115). Furthermore, the rapamycin, an classical mTOR inhibitor, could decrease the activity and expression of LDH, resulting in reduced lactate concentration (116, 117). Drugs targeting mTOR, such as rapamycin, can inhibit epileptic seizures in type II focal cortical dysplasia (FCD II) (118). Therefore, LDH may be a promising potential target for the treatment of epilepsy, and these discoveries have contributed to broaden our understanding of epilepsy and to develop new therapies.

### Dietary Therapy in Epilepsy

The history of dietary therapy for epilepsy is quite long. The ketogenic diet is a high-fat and low-carbohydrate diet structure and is considered the oldest dietary therapy initially used to treat epilepsy (119). Its effectiveness in the treatment of epilepsy, especially for some types of intractable epilepsy in children has been demonstrated (120); compared to children randomized to usual care, children randomized to KDs were three times more likely to attain seizure freedom and nearly six times more likely to attain a 50% or greater reduction in seizure frequency (121). Subsequently, various dietary options for epilepsy were developed, including the medium-chain triglyceride ketogenic diet (MCT-KD), modified Atkin's diet (MAD), and low glycemic index treatment. These modified ketogenic diets have an efficacy close to that of the classical ketogenic diet (122). In addition, diet therapy was also evaluated for adjuvant treatment of obesity, type 2 diabetes, and some cancers (123–125). Moreover, diet therapy was attempted in a wide variety of neurological diseases, including Alzheimer's disease, multiple sclerosis, Parkinson's disease, and brain tumors (126), showing that it is widely potential. However, the most widely used and effective field of ketogenic diet is in the treatment of epilepsy.

**Figure 5:**
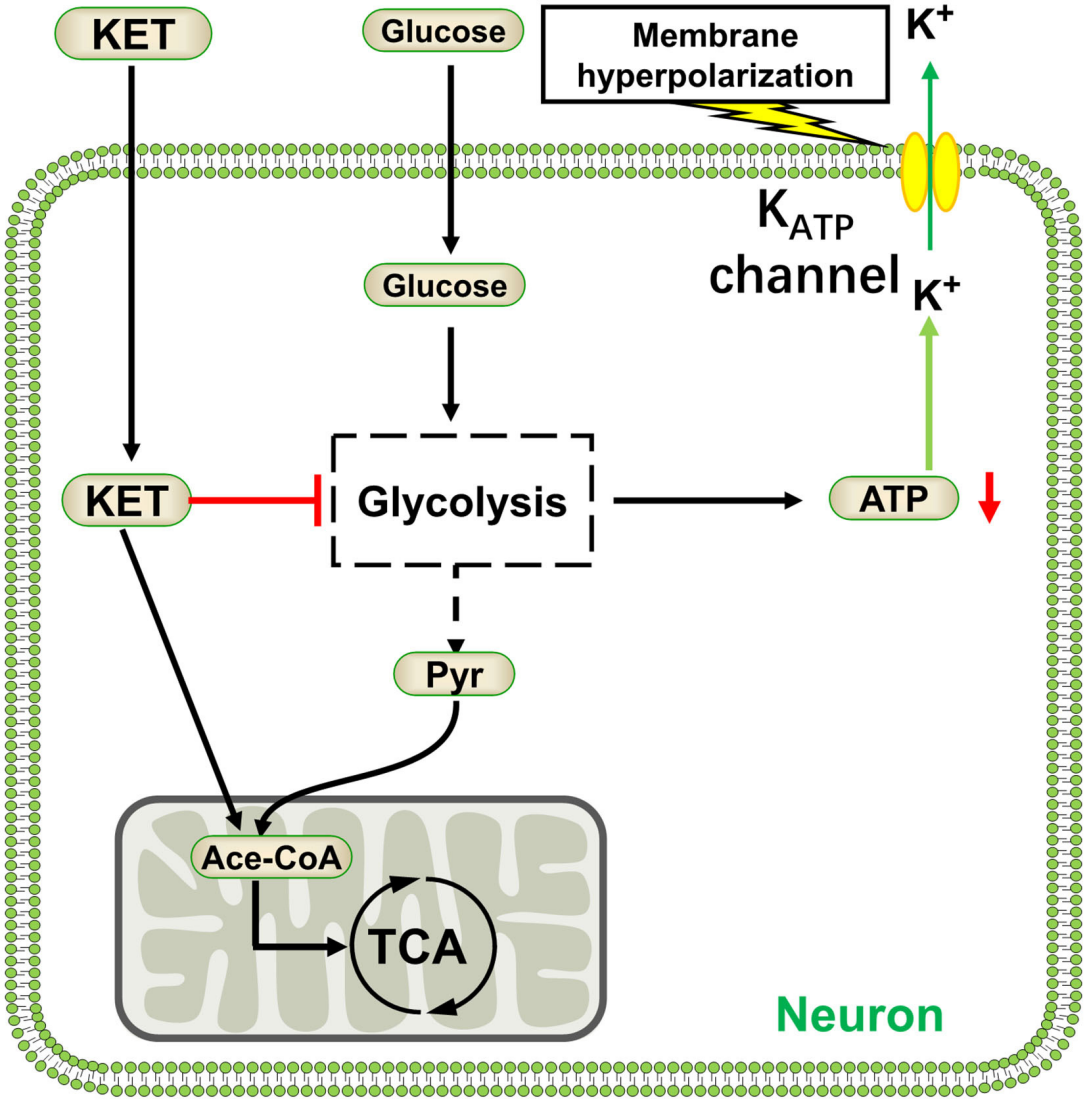
Ketone bodies activate KATP channels by inhibiting glycolysis. KET, ketone body; Pyr, pyruvate; Ace-CoA, acetyl coenzyme A.

As the name implies, ketogenic diet can produce ketone bodies *in vivo*: acetoacetic acid, beta-hydroxybutyric acid, and acetone. Studies have also shown that ketone bodies may directly exert pharmacological effects (127). The ketone body acetoacetate inhibited the transport of glutamate into synaptic vesicles by vesicular glutamate transporter 2, thus decreasing glutamate release into the synaptic cleft (128). Glutamate is a major excitatory amino acid in the brain; its signaling is inhibited by ketone bodies potentially reducing neuronal excitation and epilepsy. Conversely, ketone bodies can reduce the expression of adenosine kinase and enhance adenosine A1 receptors (A_1_R) and mediate the signaling pathway activated by adenosine (129), A_1_R activation showed anticonvulsant effects in mice and rats (130). Other studies have reported that the antiepileptic effect of the KD was due to metabolic changes caused by the conversion of energy substrates. When converting from a normal diet to a ketogenic diet, blood glucose levels decreased and ketone body concentrations significantly increased (131). At this time, the main energy substrates of the brain were converted from blood glucose to ketone bodies. The ATP production of neurons was derived from glycolysis decreases, which may lead to K_ATP_ channel activation, neuronal cell-membrane hyperpolarization, and excitability decrease (132) (Figure 5). Even in the presence of sufficient glucose, ketone bodies could inhibit neuronal firing through opening the K_ATP_ channels (133).

Fats in the traditional ketogenic diet are mainly long-chain triglycerides. To improve the traditional ketogenic diet, an MCT KD derived from the traditional ketogenic diet was first proposed in 1971 (134). The antiepileptic effect is similar to that of the traditional ketogenic diet (135, 136). The novelty is that the therapeutic effect of MCT KD may be due to the decanoic acid produced by the decomposition of medium-chain triglycerides rather than by ketone bodies. Decanoic acid has a strong direct inhibitory effect on glutamate receptor α-amino-3-hydroxy-5-methyl-4-isoxazolepropionic acid (AMPA) (137). The AMPA receptor is an excitatory ionotropic glutamate receptor mediating the bulk of the generation of excitatory post-synaptic potentials (EPSPs). EPSPs are responsible for synchronous discharges in epileptic foci, and thus AMPA receptors are critically important in the spread of epileptic discharges across brain regions (138). A highly selective AMPA receptor antagonist, perampanel (Pyramidopane, trade name Fycompa), has been promoted in the United States and the European Union as an antiepileptic drug (139). Decanoic acid produced by MCT KD directly antagonizes AMPA receptors and produces antiepileptic effects in animals, broadening our understanding of the possible antiepileptic mechanism of the ketogenic diet.

Diet therapy is an essential metabolic regulation for patients with epilepsy (95). Although dietary therapy has not fundamentally changed the status quo of epilepsy treatment, it still provides an alternative and beneficial treatment option. More importantly, it may provide inspiration for novel treatments for epilepsy.

## Status and Future of Antiepileptic Drugs

Drug therapy plays a dominant role in epilepsy treatment. Surgical treatment can be used for epilepsy caused by definite etiology and lesions, and diet therapies are usually used for children with intractable epilepsy. Some new therapies are emerging, such as neuromodulation therapy (140). However, the overall effect is not satisfactory; epilepsy cannot be effectively controlled in approximately one-third of patients. Although the new drugs are more advantageous in terms of safety and side effects, this does not appear to improve the seizure control rate (141). Antiepileptic drugs have been developed to the third generation, and more than 20 types of drugs have been used in the clinic. However, the targets or mechanisms of existing drugs are mainly focused on inhibiting excitatory NMDA receptors, voltage-dependent ion-channels, or enhancing GABAergic activity (142) (Table 1). Levetiracetam and brivaracetam bring us to a whole new antiepileptic target-synaptic vesicle protein 2A (SV2A). They display a high and selective affinity for SV2A in the brain and thus inhibit synaptic transmission, which may have antiepileptic effects (143, 144). However, the precise mechanism by which levetiracetam and brivaracetam exert their antiepileptic activity is unknown. Another marketed antiepileptic drug, stiripentol, which came on the market in 2002, was considered a GABA potentiation and sodium channel blocker (145). Targets for epilepsy treatment appear to lack diversity and innovation. However, stiripentol was proven to be a lactate dehydrogenase inhibitor in 2015. Its antiepileptic effect might be partly due to its metabolic-related enzyme inhibitory effect (13). This provided strong evidence for epilepsy controlled by metabolic modulation. In addition, 2-deoxyglucose, as a glycolysis inhibitor, has shown promising anti-epileptic effects in many pre-clinical studies (146), highlighting the prospect of controlling epileptic seizures by regulating metabolism. Research on the treatment of epilepsy has been confined to certain types of targets, such as ion channels and excitatory or inhibitory receptors of certain neuronal membranes for a long time. Epilepsy therapy calls for new targets, and metabolic pathway-related targets deserve more attention.

**Table 1:** Mechanisms of commonly used antiepileptic drugs.

**AEDs**	**Main mechanisms of action**
Carbamazepine, Oxcarbazepine, Lamotrigine, Phenytoin, Zonisamide, Rufinamide, Eslicarbazepine acetate	Sodium channel actions
Gabapentin, Pregabalin	Calcium channel blocker
Ethosuximide	T-type calcium channel blocker
Retigabine (ezogabine)	Potassium channel activator
Phenobarbital, Primidone, Diazepam, Clonazepam, Clobazam	GABA potentiation
Perampanel	Glutamate (AMPA) receptor antagonist
Stiripentol	GABA potentiation and Sodium channel blocker
Levetiracetam, Brivaracetam	SV2A modulation
Valproate, Felbamate, Topiramate	Multiple: GABA potentiation Glutamate receptor (NMDA)inhibition Sodium channel and Calcium channel blockade

*Mechanism was reviewed in (142) except for brivaracetam (143)*.

## Conclusion

Metabolism is the basis of brain function. For a long time, epilepsy has been regarded as a disease characterized by overexcitation of neural networks. The high metabolism of neurons provides energy for the hyperexcitation of neural networks. Neuroimaging and neurobiochemical findings have also confirmed that epileptic foci have high metabolic characteristics, and some metabolic disorders will lead to epileptic seizures. These fully illustrate the close relationship between epilepsy and metabolism. Epilepsy has even been defined as a metabolic disease. Recognizing that the ketogenic diet, an ancient dietary therapy for epilepsy, may be essentially a metabolic regulation method, suggesting that epilepsy may be controlled by targeted metabolism pathways. Drugs that reduce metabolisms, such as 2-deoxyglucose and stiripentol, have antiepileptic effects, showing the prospects of metabolic control of epilepsy. Therefore, the relationship between metabolism and epilepsy may be bi-directional. Hypometabolism could initiate convulsions or epilepsy, such as hypoglycemia, GLUT1 deficiency, and mitochondrial dysfunction. Supplementing neuronal energy is an effective treatment in such conditions. However, there is a relatively high metabolic and energy-consuming state in the epileptic seizure period caused by various primary pathological changes; thus, reducing the metabolic level during epileptic seizures is helpful for epilepsy control. Epilepsy therapy calls for more effective new targets, and the metabolic pathway may be a promising alternative. Epilepsy control by metabolism regulation may provide the means to explore new targets of epilepsy treatment. Thus, the treatment of epilepsy by metabolic modulation is a rising panacea for epilepsy. In the future, we need to further elucidate the metabolic processes taking place in the highly complex brain as well as metabolic changes under pathological conditions in order to locate key targets.

## Author Contributions

YF put forward the conception of the review and wrote the manuscript. RS participated in the proposal of the concept and revised the manuscript. JW and ZS proposed suggestions for revision. All authors contributed to the article and approved the submitted version.

## Conflict of Interest

The authors declare that the research was conducted in the absence of any commercial or financial relationships that could be construed as a potential conflict of interest.
